# The Effect of Aerobic Exercise on Brain-Derived Neurotrophic Factor in People with Neurological Disorders: A Systematic Review and Meta-Analysis

**DOI:** 10.1155/2017/4716197

**Published:** 2017-09-19

**Authors:** Christopher P. Mackay, Suzanne S. Kuys, Sandra G. Brauer

**Affiliations:** ^1^Faculty of Health and Behavioural Sciences, School of Health and Rehabilitation Sciences, The University of Queensland, Brisbane, Australia; ^2^Faculty of Health Sciences, School of Physiotherapy, Australian Catholic University, Brisbane, Australia

## Abstract

**Objective:**

To determine the effect of aerobic exercise on brain-derived neurotrophic factor (BDNF) levels in people with neurological disorders.

**Data Sources:**

Six electronic databases (CINAHL, PubMed, Cochrane, PsycINFO, SportDiscus, and Web of Science) were searched until the end of December 2016.

**Study Selection:**

Experimental or observational studies of people with neurological disorders who undertook an exercise intervention with BDNF as an outcome measure. The search strategy yielded 984 articles.

**Data Extraction:**

Study data were independently extracted from each article. Methodological quality of studies was assessed using the Physiotherapy Evidence Database (PEDro) scale. A meta-analysis was planned based on the assessment of predetermined criteria.

**Data Synthesis:**

Eleven articles were included. Studies employed either a program of aerobic exercise, a single bout of aerobic exercise, or both. A meta-analysis of studies comparing a program of aerobic exercise against usual care/nil therapy showed a large effect (SMD: 0.84, 95% CI 0.47–1.20, *p* < 0.001) in favour of aerobic exercise to increase levels of BDNF. Findings for a single bout of aerobic exercise were mixed. Quality of studies was low (PEDro average score 4.3/10).

**Conclusions:**

A program of aerobic exercise may contribute to increased levels of BDNF in neurological populations.

## 1. Introduction

Aerobic exercise is a training intervention that has produced a variety of positive impacts in people with neurological disorders. For example, after a program of aerobic exercise, individuals with stroke and Parkinson's disease have shown improvements in walking [1–3], functional ability [4, 5], and motor performance [6] in addition to gains in cardiorespiratory fitness [7]. Several mechanisms have been proposed to explain the positive impacts of aerobic exercise. These include increased cerebral blood flow, changes to neurotransmitter release, structural changes in the central nervous system, and altered arousal levels [8]. A more recent proposal indicates neurotrophic factors, specifically brain-derived neurotrophic factor (BDNF) as a possible agonist in facilitating improved motor performance [9, 10].

BDNF is a member of the neurotrophin family of proteins found in the peripheral and central nervous systems, known to play an important role in neuron development, plasticity, differentiation, and survival [11–13]. Aerobic exercise is proposed to induce the expression of BDNF throughout the central nervous system, which in turn, can enhance synaptic plasticity [9]. A recent review and meta-analysis of 29 studies investigating the effect of exercise on BDNF in healthy humans [14] found that a single session of aerobic exercise significantly increases BDNF levels immediately postexercise demonstrating a moderate effect (Hedges' *g* = 0.46). Furthermore, in the same review, a program of aerobic exercise was shown to significantly increase resting levels of BDNF, with a small effect size reported (Hedges' *g* = 0.27). These findings provide evidence that aerobic exercise has a significant impact on BDNF levels in healthy humans.

People with neurological disorders have potential to harness neuroplasticity to facilitate recovery of motor performance. In a rat model of brain embolism, studies have reported that a bout of aerobic exercise has resulted in an increase in levels of BDNF, with parallel increases in sensorimotor learning of a maze task [15] and skilled reaching [16], and improved memory [16]. Furthermore, in rat models of stroke, skilled motor performance is impaired when BDNF production is disturbed via pharmacological intervention [16] and facilitated when it is enhanced [17]. There are growing numbers of studies aiming to elucidate the impact of aerobic exercise on BDNF levels in people affected by neurological disorders. A synthesis of recent evidence may assist to understand the extent to which aerobic exercise may upregulate BDNF in these populations. This is likely to be of particular significance in these populations given the (arguably) greater potential for relearning and impact on potential for neuroplasticity. Therefore, this review aims to investigate the effects of aerobic exercise on BDNF levels in neurological populations, both (1) the effect of a single bout of aerobic exercise on BDNF levels immediately postexercise and (2) the effect of a program of regular aerobic exercise on resting BDNF levels.

## 2. Methods

The preparation and reporting of this review were undertaken according to the Preferred Reporting Items for Systematic Reviews and Meta-Analyses (PRISMA) statement [18] and registered with the international prospective register of systematic reviews (PROSPERO) [19].

### 2.1. Literature Search

**Figure 1:**
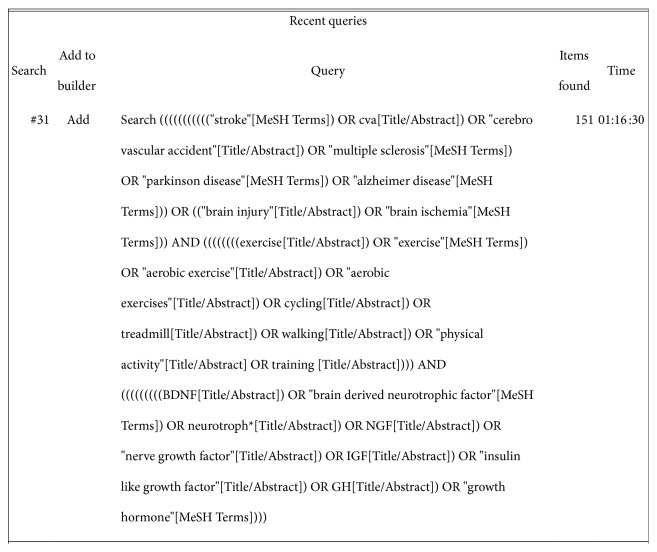
Database search history—PubMed.

Electronic databases were systematically searched from inception until December 31st, 2016, with the following search term categories used in combination: *neurotrophins (e.g., BDNF, nerve growth factor), exercise (e.g., treadmill, cycling), and neurological condition/event (e.g., stroke, multiple sclerosis).* An example is provided in Figure 1. Searched databases included CINAHL, PubMed, COCHRANE, PsycINFO, SportDiscus, and Web of Science. Reference lists of included studies and relevant reviews were also screened for eligible articles. No language restrictions were imposed on the retrieved articles.

### 2.2. Eligibility Criteria

Study title and abstract of retrieved articles were screened by two independent reviewers. Full-text articles were reviewed and included for analysis based on the following eligibility criteria: (1) human studies, (2) investigating a neurological population with a motor impairment, (3) BDNF in serum or plasma as an outcome measure, (4) used an experimental or observational study design, and (5) included an exercise intervention. Studies were excluded based on the following criteria: (1) animal studies, (2) no measurement of BDNF in serum or plasma, (3) review studies, (4) no neurological population, (5) no exercise intervention, and (6) duplicates. Any disagreements between reviewers were resolved by consensus.

### 2.3. Quality Assessment

Studies were appraised for methodological quality and risk of bias according to the Physiotherapy Evidence Database (PEDro) criteria. The PEDro criterion defines quality using an 11-item scale (scored/10 from criteria 2–11), with reported high content validity [20] and fair to good reliability [21]. This scale is a valid measure of the methodological quality of clinical trials [22]. Two authors independently assessed the quality of each paper with any disagreements resolved by consensus.

### 2.4. Data Extraction

Study data were extracted from each article including study design, population (condition), participant information (e.g., gender, age), details of the intervention, and study outcomes. Serum BDNF levels (means and standard deviations) were extracted from the studies that published this information. Where data were reported in another format, study authors were contacted to provide this data. A meta-analysis was planned based on the assessment of the following predetermined criteria: (i) Is there a clinically homogenous group of studies within the included studies? (ii) Is there a statistically homogenous group of studies based on a chi-squared test? (iii) Is relevant data reported in order to conduct a meta-analysis (i.e., means and standard deviations for experimental and control groups)? Using a chi-squared test, it was planned that a fixed-effects model of analysis would be used if heterogeneity between studies was low (<40%) and a random effects model would be used if heterogeneity was high (75–100%) [23]. Standardised mean differences (SMD) and confidence intervals (CI) were calculated in order to determine effect size. By convention, an effect size of 0.2, 0.5, and 0.8 is considered small, medium, and large, respectively [24]. All metadata analyses were conducted using the Cochrane Review Manager software, RevMan v. 5.3. (http://ims.cochrane.org/revman/download).

## 3. Results

### 3.1. Included Studies

**Figure 2:**
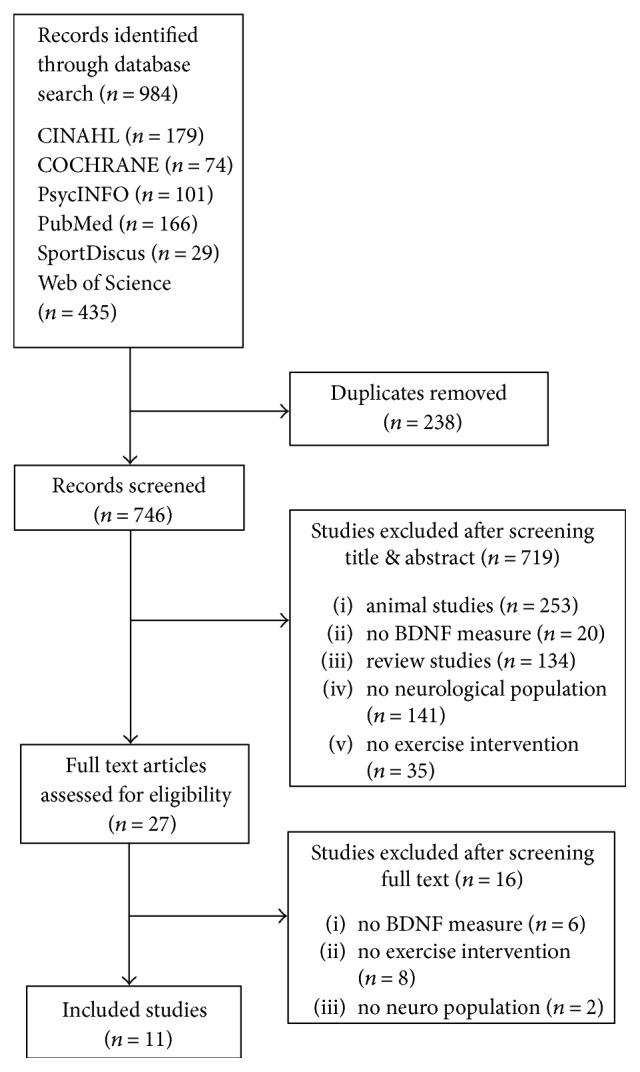
A flow diagram of the systematic review literature search.

Eleven original articles were included for analysis [25–35]. The search strategy yielded a total of 984 articles of which 238 were duplicates, leaving 746 articles that were screened for eligibility (Figure 2). A total of 719 articles were excluded based on title and abstract. Twenty-seven (27) articles met the eligibility criteria, and full-text copies were retrieved. On review of the full text, a further sixteen (16) articles were excluded due to inability to satisfy all eligibility criteria, leaving eleven articles to be included for analysis. One study was reported in two publications, with the primary outcomes reported in one paper [36], and the details of the methods in another [26]. All of the included studies were published after 2003, with eight published after 2012.

A total of 303 participants were included in the studies (Table 1). The mean age (±SD) of participants ranged from 39 ± 9 to 77 ± 8 years. Females represented 55% of the participants. Sample size of the included studies was comparatively small, averaging 27.5 with only one study having more than 50 participants [26]. Six studies investigated exercise in people with multiple sclerosis [26, 28, 29, 32, 34, 35], three with Parkinson's disease [25, 31, 33], and two in stroke [27, 30].

A majority of studies (*n* = 10) [25–31, 33–35] investigated a program of aerobic exercise over a period of weeks, ranging from three to twenty-four weeks in duration, and five studies [26, 28, 29, 32, 34] investigated a single bout of exercise. A program of exercise is defined here as multiple sessions of exercise, planned regularly over a period of weeks. Most studies employing a program of aerobic exercise did so three times per week for approximately 30 minutes in duration. A majority of studies (10 of 11) used a bike ergometer as the aerobic exercise intervention, with a moderate intensity of 60% maximal oxygen uptake—VO_2_ max—used most frequently [26, 29, 32]; otherwise, a variety of exercise intensities were reported. Other modes of aerobic exercise included treadmill walking (with or without body weight support) and immersed cycling underwater, with additional types of exercise compared including strength training, balance work, stretching, and electrical stimulation (Table 1).

BDNF was the primary outcome measure of interest in this review. All studies measured BDNF via collection of peripheral blood as serum. Ten studies collected blood from participants at rest, generally in the morning prior to an exercise session, in order to measure basal levels of BDNF. Five studies collected blood postexercise to measure the immediate effect of exercise on BDNF. Four of the included studies took measures of blood both at rest and postexercise.

### 3.2. Quality Assessment

The average quality rating of the included studies using the PEDro criteria was low, 4.3 out of a possible 10 (Table 2). The PEDro criteria for rating quality are designed to assess randomised controlled trials (RCTs), and of the eleven studies included in this review, five were RCTs [26, 28, 31, 34, 35] and had the highest quality rating scores. Criteria 11 (point measures and measures of variability), 10 (reporting between group results), and 8 (key outcome in >85% of subjects) were fulfilled in 64–100% of trials, and only two studies performed blinded assessment. As the participants in each study were aware of whether they received an intervention, none of the included studies were able to blind patients or therapists. Therefore, no studies were able to satisfy these two criteria on the quality rating scale.

### 3.3. Effect of a Program of Aerobic Exercise

Ten studies evaluated the change in resting BDNF following a program of aerobic exercise with a duration ranging from 6.5 to 60 hrs (Table 1). Descriptively, five studies showed a statistically significant increase in resting BDNF levels following a program of aerobic exercise. Equally, five studies showed no significant change in BDNF levels at the end of the program. Of those studies that showed no significant difference in BDNF, the average volume of hours spent exercising (over the length of the program) was 12.9 ± 3.9 hours (median 12 hours). Comparatively, in the studies that showed a statistically significant change in BDNF, the average volume of hours spent exercising was 20.6 ± 20 hours (median 12 hours).

**Figure 3:**
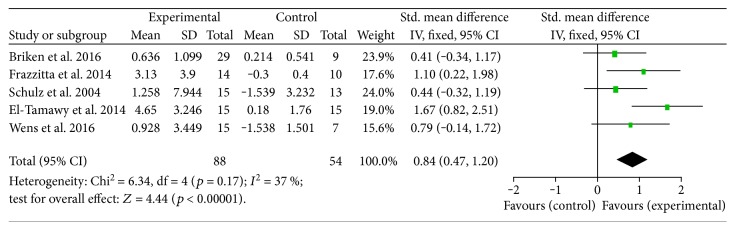
Meta-analysis of RCT/CT studies investigating a program of aerobic exercise (experimental group) versus usual care/nil therapy (control group).

Five studies were identified for inclusion in a meta-analysis based on the statistical and clinical homogeneity of studies (Figure 3). These studies employed a randomised controlled or controlled trial study design, allocated participants to an aerobic exercise group or a control group that received no intervention/usual care, and included people with MS (2 studies), Parkinson's disease (2 studies), and stroke (1 study). Results show a statistically significant and large effect of aerobic exercise on BDNF in favour of the experimental (aerobic exercise) group, compared to the control group (usual care/nil therapy) (SMD: 0.84, 95% CI 0.47 to 1.20, *p* < 0.001), with a low observed heterogeneity between studies (*I*^2^ = 37%, *p* < 0.05).

### 3.4. Effect of a Single Bout of Aerobic Exercise

Five studies investigated the effect of a single bout of aerobic exercise on BDNF levels immediately postexercise in people with neurological conditions (Table 1). Two studies included a control group of exercising healthy participants [29, 32], and three were RCTs [26, 28, 34]. Of the studies investigating a single bout of aerobic exercise, three showed a significant increase in BDNF immediately postexercise, and two showed no change (Table 1). In relation to intensity of exercise, two studies [26, 28] exercised participants at a high or maximal intensity (until exhaustion or symptom-limited maximum) with both studies showing a statistically significant increase in BDNF immediately postexercise, demonstrating a moderate effect (ranging from 0.45 to 0.48). The remaining three studies exercised participants at a low–moderate intensity with only one of these studies showing a statistically significant increase in BDNF immediately postexercise.

## 4. Discussion

This review and meta-analysis suggest that a program of aerobic exercise can increase levels of BDNF in people with a neurological disorder when compared to usual care or nil therapy. An upregulation of BDNF is considered desirable as it is associated with enhanced plasticity-related processes such as dendritic growth, neurogenesis, and long-term potentiation of neurons [14]. Regular aerobic exercise may therefore have the potential to convey benefits resulting from enhanced neuroplasticity in the affected brain.

This positive finding of a program of aerobic exercise upregulating BDNF supports a recent systematic review and meta-analysis [14] of 15 studies investigating the effect of a program of aerobic exercise on BDNF levels in predominantly healthy individuals. The size of the effect in the current study is larger in comparison to that found in healthy adults following regular exercise (Hedges' *g* = 0.27).

Evidence for an effect of aerobic exercise on BDNF levels has also been confirmed and consistently reported in animal studies poststroke, with aerobic exercise leading to increased BDNF with a concomitant improvement in function, specifically in a maze running task [9], improvement in skilled reaching [16], and enhanced visual and tactile paw placement [37]. Successful use of aerobic exercise as a primer has also been shown in animals with significantly improved performance in a reaching task when running was performed directly prior [38]. Experimental studies of Parkinson's disease in animals have also shown an upregulation in BDNF in response to aerobic exercise with related improvement in symmetrical forelimb movement [39] and improved balance [40]. Similar links between raised BDNF levels and improvements in motor function in human neurological populations have yet to be established.

But, approximately half of the studies reported in this review did not find a positive effect of aerobic exercise on BDNF levels. Many factors may have contributed to no effect. This may include, as others have indicated [35], that variation in intensity or dose of exercise can alter the effect on BDNF levels. In the current review, a significant increase in BDNF levels was found in studies where on average, 20 hours of aerobic exercise was performed. Comparatively, in the studies that showed no change in BDNF levels, the average volume of hours spent exercising was 12.9 ± 3.9 hours. This result lends support to the suggestion that varying the dose of exercise may impact levels of BDNF, as suggested by Knaepen et al. [41]. In their systematic review investigating both healthy individuals and those with a disability or disease, results showed that programs of aerobic exercise performed 2-3 times/week had no significant effect on BDNF levels but that the same intervention performed 4–7 times/week significantly increased levels of BDNF.

As well as a program of exercise, a single bout of aerobic exercise was investigated in this review with results suggesting no significant impact on levels of BDNF. This finding is contrary to a recent meta-analysis of aerobic exercise in healthy adults (*n* = 14 studies), where a significant increase in BDNF was found after a single exercise bout (Hedges' *g* = 0.46, *p* < 0.001) [14]. The inability to draw firm conclusions about a single bout of aerobic exercise in this review may relate to the small number of papers available, or to the varying intensity of training seen across studies. In the current review, five studies investigated the effect of a single bout of aerobic exercise on levels of BDNF. Two of these studies performed a single bout of aerobic exercise at a high intensity (maximal/until exhaustion) [26, 28], with both finding a significant increase in levels of BDNF postexercise. Of the three studies investigating a single bout of aerobic exercise performed at a low–moderate intensity, only one showed a significant change in levels of BDNF. Other reviews in healthy adults have also suggested that exercise intensity should be considered as a potential moderator of the expression of BDNF [14, 41].

So, the intensity of exercise may influence the upregulation of BDNF, and the small number of studies available may limit conclusions drawn, but it is also plausible that a single bout of aerobic exercise has minimal to no immediate effect on BDNF levels in neurological populations. As mentioned, this is contrary to healthy data and may represent an important difference between the effect of a single bout of aerobic exercise and a program of exercise in neurological populations. One explanation for this difference may be that the increase in BDNF seen with a program of exercise in neurological populations is due to a cumulative dose of regular exercise. In their systematics review, Szuhany et al. [14] reported that regular exercise had double the effect on BDNF levels in populations with psychiatric disorders (e.g., depression) than healthy adults (mean effect size 0.40 for psychiatric versus 0.19 for healthy). A similar effect may be occurring in neurological populations and may explain why a greater effect is found after a program of exercise than a single isolated exercise bout. However, due to the small number of studies and variation between studies, further high-quality trials are recommended to clarify the effect of a single bout and a program of aerobic exercise on BDNF levels in neurological populations.

Measured levels of BDNF at baseline varied across studies of the same population, between healthy control groups and between neurological populations and healthy controls. Differences in basal levels of BDNF are not unexpected and may relate to age, sex, diurnal fluctuations, diet, and disorders of the metabolic or immunological systems [14, 41–44]. Another well-founded explanation for variation in BDNF levels relates to a polymorphism on the BDNF gene (Val66Met) which is common in humans [45]. Presence of this polymorphism is associated with decreased activity-dependent release of BDNF, that is, there is less circulating BDNF in people who have the polymorphism [46]. Few studies undertake this genetic testing of participants in exercise intervention studies, but randomisation of study participants may minimise this effect.

### 4.1. Clinical Implications

Over the past two decades, as mortality rates have declined, the population has aged, with disability becoming increasingly important [47]. As the global burden of disease shifts away from years of life lost due to premature death, towards years of life lost due to disability, there is an increasing need to investigate and discover new ways to reduce this disability. This is especially the case for individuals with a neurological disorder, as they (as a group) represent one of the highest contributors to the burden of disease and disability globally [48].

There is a growing body of research highlighting that the intensity of activity in rehabilitation settings is often inadequate for therapeutic gains and could contribute to disability and a slow rate of recovery [49, 50]. There is an urgent need to investigate methods to enhance the gains made by rehabilitation as many service delivery models only permit a short period of active rehabilitation. Interventions to “prime” the brain to make it more receptive or enhance the effect of training need to be investigated to enhance both motor and cognitive function and lead to greater improvements in the recovery of people with neurological disorders due to rehabilitation. The findings of this review suggest that regular aerobic exercise may enhance levels of BDNF in neurological populations. The magnitude of this effect, though, may relate to the intensity or dose of exercise, highlighting the need for future studies to establish a potential dose-response relationship.

### 4.2. Study Limitations

The findings from this review implicate regular aerobic exercise as a contributor to increased BDNF levels; however, limitations to the evidence should be considered. We found that the overall quality of the included papers was low when measured with the PEDro scale. Higher quality trials are needed to confirm these findings using a randomised design and employing strategies to minimise the influence of confounding factors. Different neurological populations were considered in this review which have different underlying pathology. The impact of BDNF levels on motor skill relearning may differ between and quite possibly within populations. The effect of publication bias should also be considered—only published studies were included; studies with negative results are often not published.

## 5. Conclusions

This review provides evidence that aerobic exercise has a positive impact on levels of BDNF in neurological populations, as measured by peripheral blood. Including regular aerobic exercise as a component of rehabilitation in a neurological setting may assist to increase BDNF levels, potentially leading to the enhancement of neuroplasticity and facilitating improved motor performance. Further, high-quality trials are required to confirm the results of this review, with specific attention should be paid to study design and factors that may influence BDNF results.

## Figures and Tables

**Table 1:** Study characteristics.

Study	Population	Design, quality	Groups	Program duration	Dose, intensity	Mode	Time of measure	Postprogram outcome	Single bout outcome
Angelucci et al. [25]	Parkinson's disease (*n* = 9)	Pre-post	PD exercise	4 weeks	40 min 5 days/wk ≤60% HRR	Treadmill & bike ergometer	At rest	BDNF NSD Pre-post *p* < 0.14	
Bansi et al. [26, 36]	Multiple sclerosis (*n* = 52)	RCT	MS aqua bike versus MS land bike	3 weeks	30 min 5 days/wk 60% VO_2_ peak	Aqua bike & land bike ergometer	At rest & postexercise	BDNF ↑ postaqua ex *p* = 0.046, NSD land *p* = 0.69	BDNF ↑ postaqua ex *p* = 0.002, NSD land *p* = 0.585
Bhasin et al. [27]	Stroke (*n* = 10)	Pre-post	Stroke exercise	8 weeks	10 min 5 days/wk	Bike ergo step ups/down	At rest	BDNF NSD	
Briken et al. [28]	Multiple sclerosis (*n* = 42)	RCT	MS exercise versus MS no exercise	9 weeks	15–45 min 2-3 days/wk 120–130% of AT	Arm ergo rower bike ergo	At rest & postexercise	BDNF NSD between groups postex *p* = 0.27	BDNF ↑ postex postex *p* < 0.001
Castellano and White [29]	Multiple sclerosis (*n* = 11), healthy controls (*n* = 11)	QE	MS exercise versus healthy exercise	8 weeks	30 min 3 days/wk 60% VO_2_ peak	Bike ergometer	At rest & postexercise	BDNF ↑ in MS at 4wks *p* = 0.04, NSD in MS at 8wks *p* = 0.10	BDNF NSD postex in MS *p* > 0.3
El-Tamawy et al. [30]	Stroke (*n* = 30)	CT	Stroke exercise versus stroke usual care	8 weeks	30 min 3 days/wk intensity not stated	Bike ergometer	At rest	BDNF ↑ postex *p* < 0.001, NSD control *p* = 0.70	
Frazzitta et al. [31]	Parkinson's disease (*n* = 24)	RCT	PD exercise versus PD no exercise	4 weeks	3 × 60 min 5 days/wk intensity not stated	Treadmill, strength & relaxation	At rest	BDNF ↑ postex *p* = 0.017, NSD control *p* > 0.05	
Gold et al. [32]	Multiple sclerosis (*n* = 25), healthy controls (*n* = 20)	QE	MS exercise versus healthy exercise	Single bout	30 min 60% VO_2_ max	Bike ergometer	Postexercise		BDNF ↑ both groups postex *p* = 0.03
Marusiak et al. [33]	Parkinson's disease (*n* = 11), healthy controls (*n* = 11)	QE	PD exercise versus healthy no exercise	8 weeks	40 min 3 days/wk 68% HR max	Bike ergometer & interval training	At rest	BDNF ↑ postex *p* = 0.035, NSD control *p* = 0.81	
Schulz et al. [34]	Multiple sclerosis (*n* = 25)	RCT	MS exercise versus MS no exercise	8 weeks	30 min 2 days/wk 75% max watts	Bike ergometer	At rest & postexercise	BDNF NSD between groups postex *p* = 0.17	BDNF NSD between groups postex *p* = 0.18
Wens et al. [35]	Multiple sclerosis (*n* = 22)	RCT	MS exercise versus MS usual care	24 weeks	20 min 2-3 days/wk BORG 12–14	Treadmill & bike ergo	At rest	BDNF ↑ postex but NSD *p* = 0.1 BDNF ↓ control *p* < 0.05	

PD: Parkinson's disease; HRR: heart rate reserve; NSD: nonsignificant difference; RCT: randomised controlled trial; ex: exercise; MS: multiple sclerosis; VO_2_: volume of oxygen; AT: anaerobic threshold; ergo: ergometer; QE: quasiexperimental; CT: controlled trial; HR: heart rate; BORG: rating of perceived exertion.

**Table 2:** Quality assessment of the included studies using the PEDro checklist^∗^.

Study	C1	C2	C3	C4	C5	C6	C7	C8	C9	C10	C11	Total^∗^
Angelucci et al. [25]	Y	N	N	N	N	N	N	N	N	N	Y	1
Bansi et al. [26]	N	Y	Y	Y	N	N	Y	Y	N	Y	Y	7
Bhasin et al. [27]	Y	Y	N	Y	N	N	N	Y	N	Y	Y	5
Briken et al. [28]	Y	Y	Y	N	N	N	N	Y	N	Y	Y	5
Castellano and White [29]	N	N	N	N	N	N	N	N	Y	Y	Y	3
El-Tamawy et al. [30]	N	N	N	Y	N	N	N	N	N	N	Y	2
Frazzitta et al. [31]	N	Y	Y	Y	N	N	Y	Y	Y	Y	Y	8
Gold et al. [32]	N	N	N	N	N	N	N	N	N	Y	Y	2
Marusiak et al. [33]	N	N	N	N	N	N	N	Y	N	Y	Y	3
Schulz et al. [34]	N	Y	N	Y	N	N	N	Y	N	Y	Y	5
Wens et al. [35]	Y	Y	N	Y	N	N	N	Y	Y	Y	Y	6

C1: eligibility criteria; C2: random allocation; C3: concealment of allocation; C4: group similarity at baseline; C5: blinding of subjects; C6: blinding of therapists; C7: blinding of assessors; C8: one key outcome obtained from 85% of subjects; C9: intention to treat analysis; C10: between group statistical comparisons; C11: point and variability measures for at least 1 key outcome. ^∗^Criteria 2–11 scored.

## Abbreviations

BDNF: Brain-derived neurotrophic factor
PEDro: Physiotherapy Evidence Database scale
SMD: Standardised mean difference
VO_2_ max: Maximal oxygen uptake
RCT: Randomised controlled trial.
